# Supplementary material for: Single-protein/RNA imaging reveals ZNF598 as a limiting factor in resolving collided ribosomes
Source: EMBO J. 2025 Aug 1;44(18):5215–32. doi: 10.1038/s44318-025-00523-z (PMC12436648; doi:10.1038/s44318-025-00523-z)
Supplement: Supplementary file 1 — Appendix [file 44318_2025_523_MOESM1_ESM.pdf]

Appendix for Single-protein/RNA imaging reveals ZNF598 as a limiting factor in resolving collided ribosomes

Ana C. De La Cruz[1,2], Garrett Tisdale[1,2], Emily Nakayama[1,2], Zhiyuan Huang[1,2], Niladri K. Sinha[3,4], Rachel Green[3,4], Bin Wu[1,2,5,6,#]

Appendix Table of Contents

Appendix Figure S1. 2
Appendix Figure S2. 3
Appendix Figure S3. 4
Appendix Figure S4. 5
Appendix Figure S5 6
Appendix Figure S6 7
Appendix Table S1. 9
Appendix Table S2. 9
Appendix Table S3. 10

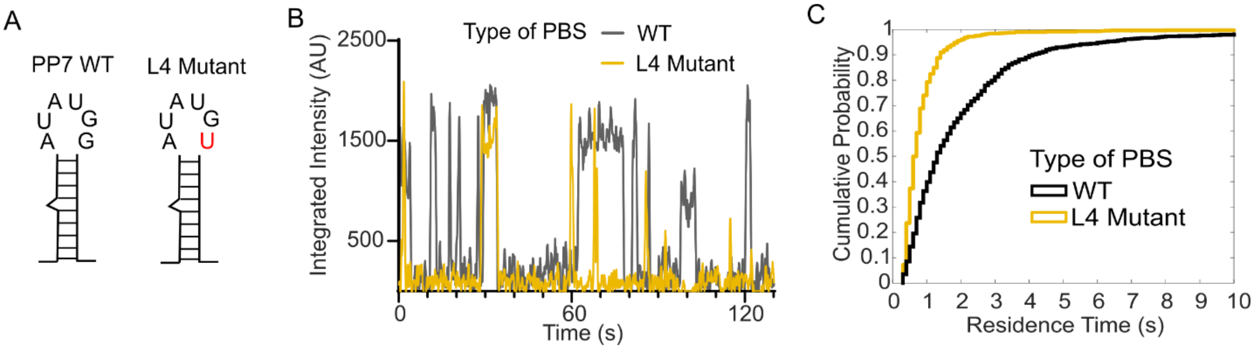

Appendix Figure S1. Binding kinetics of PCP-Halotag to WT vs L4 Mutant 1xPBS.

A. Simplified scheme of PP7 Stem loop sequence for WT vs L4 Mutant**.**

B. Example trace of tdPCP-HaloTag intensity over time for WT vs L4 1xPBS mRNAs.

C. Cumulative distribution plot of the "on" bound residence times for PCP-HaloTag to 1x PBS WT or 1xPBS L4-mutant. Two biological replicates for each condition (WT: 67, 83 tracks; L4: 107, 93 tracks).

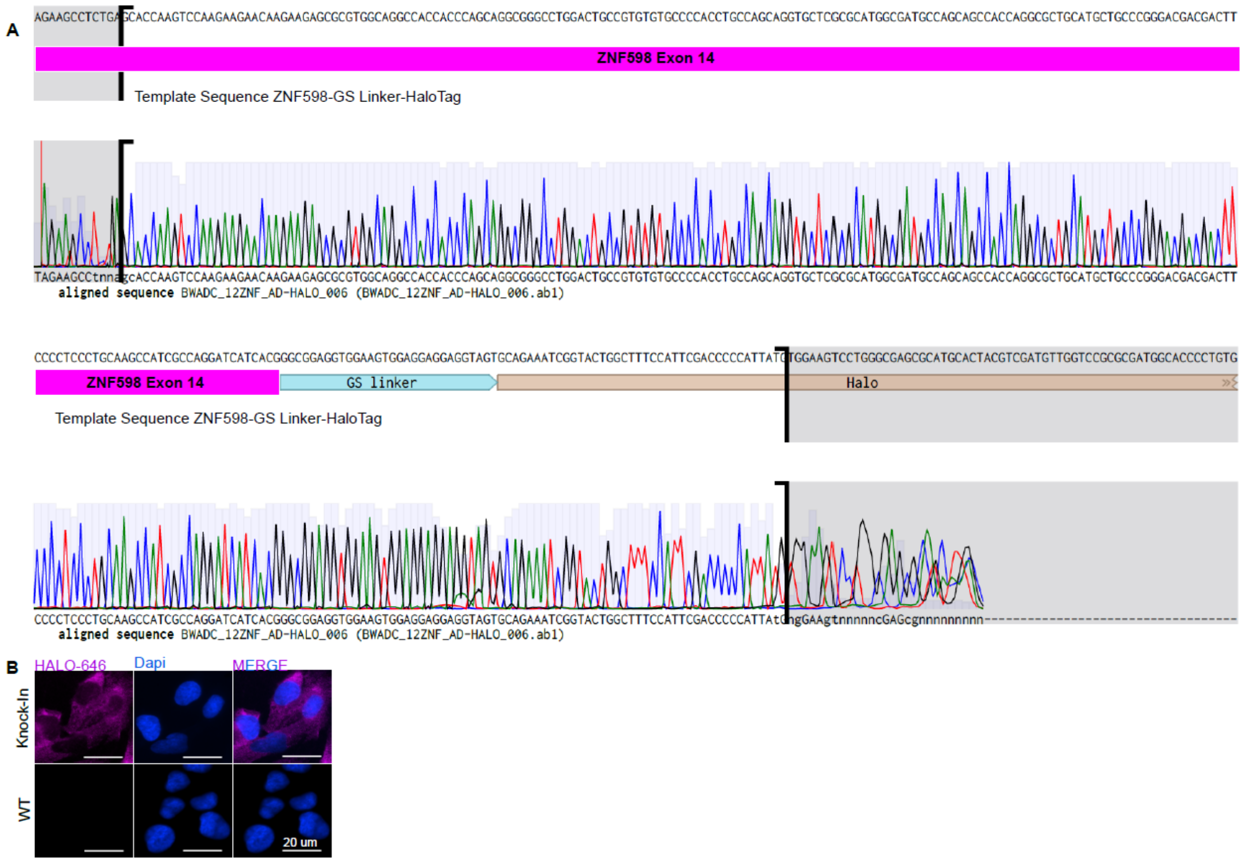

Appendix Figure S2. Validation of Endogenous ZNF598 Tagged with HaloTag in U-2 OS Cells, associated with Figure 2.

A. Sanger Sequencing results of Znf598 locus in exon 14 after GS-HaloTag insertion.

GS: Glycine-Serine linker.

B. Representative fluorescent images of wild-type (WT) and knock-in U-2 OS cell lines stained with JFX646 Halo dye.

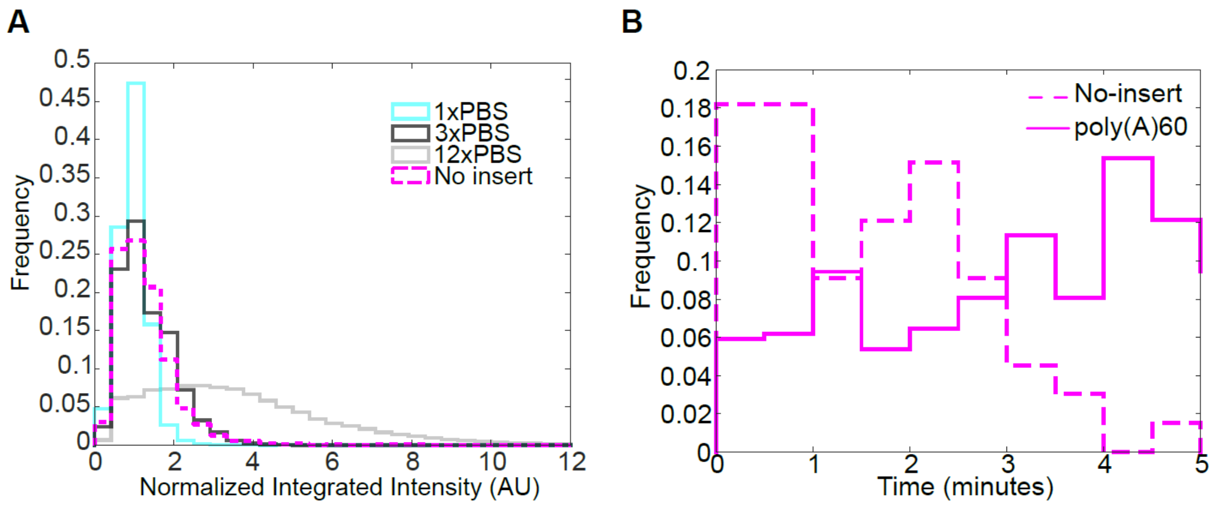

Appendix Figure S3. Intensity and residence time of ZNF598 on single translating mRNAs, associated with Figure 3.

A. Normalized integrated intensities of ZNF598-HaloTag colocalized to translating mRNA. The histogram of 1x (cyan), 3x (black) or 12xPBS (gray) mRNAs from Fig. 1F were reproduced here for comparison. Data was compiled from two independent experiments. (1x: 148, 213, 193; 3x: 229, 266, 270; 12x: 212, 404, 194; No-insert: 39, 27 tracks)

B. Distribution of the time ZNF598-HaloTag is bound to a single translating mRNA.

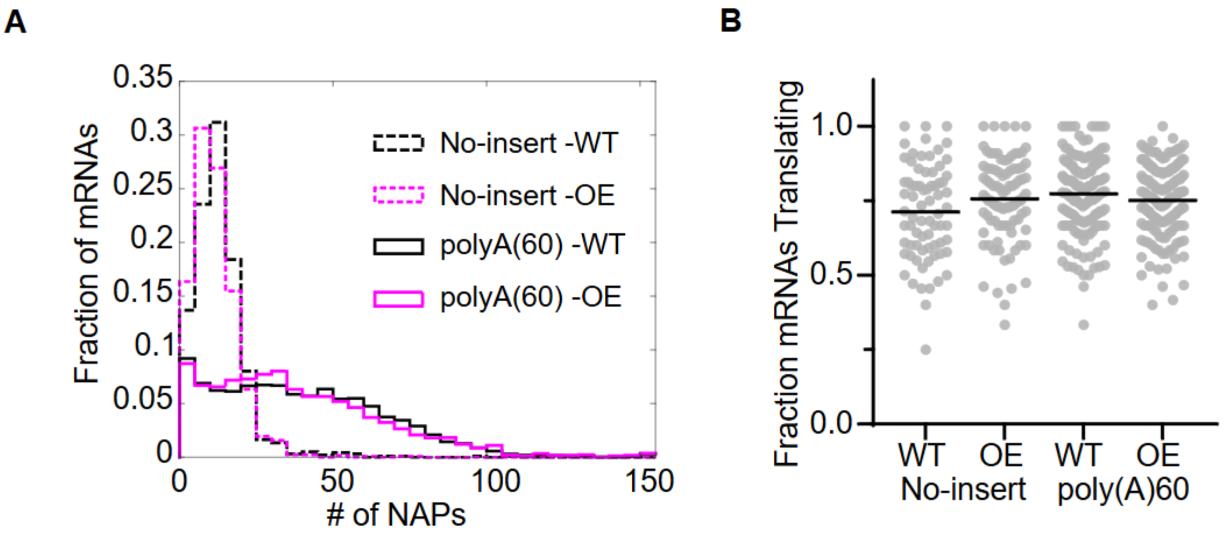

Appendix Figure S4. Wild-type versus Overexpression of ZNF598 cell line have similar ribosome load.

A. Quantification of the number of Nascent Peptides (NAPs) per mRNA in wild-type (mean: no-insert=13.06; poly(A)60=39.69) vs overexpression of ZNF598 (mean: no-insert=11.65; poly(A)60=38.73) cells. p-value for comparison of WT vs OE for No-insert: 0.9695. p-value for comparison of WT vs OE for poly(A)60: 0.2719. P values calculated by two-sample t test after correction of distribution skewness (see methods for details)

B. Fraction of mRNAs actively translating. Each dot represents one cell; black lines indicate mean. p values calculated by two-sample t test. Data compiled from two independent experiments. mRNAs calculated to have >150 ribosomes are included in the rightmost bin. 73–165 cells; 1,391–4393 mRNAs per condition. p value for comparison of Fraction of mRNA translating in WT to OE for no-insert:0.3328; p value for comparison of Fraction of mRNA translating in WT to OE for poly(A)60: 0.5522. p values calculated by two-sample t test.

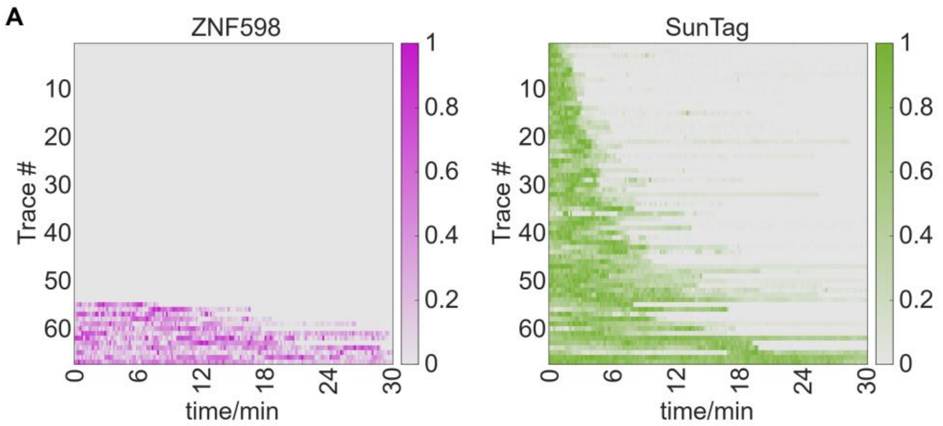

Appendix Figure S5. ZNF598-pre-bound mRNAs have slower runoff rates than unbound mRNAs.

A. Composite of normalized ZNF598 tracks and their corresponding no-insert SunTag Runoff trajectories (4 cells; 67 tracks).

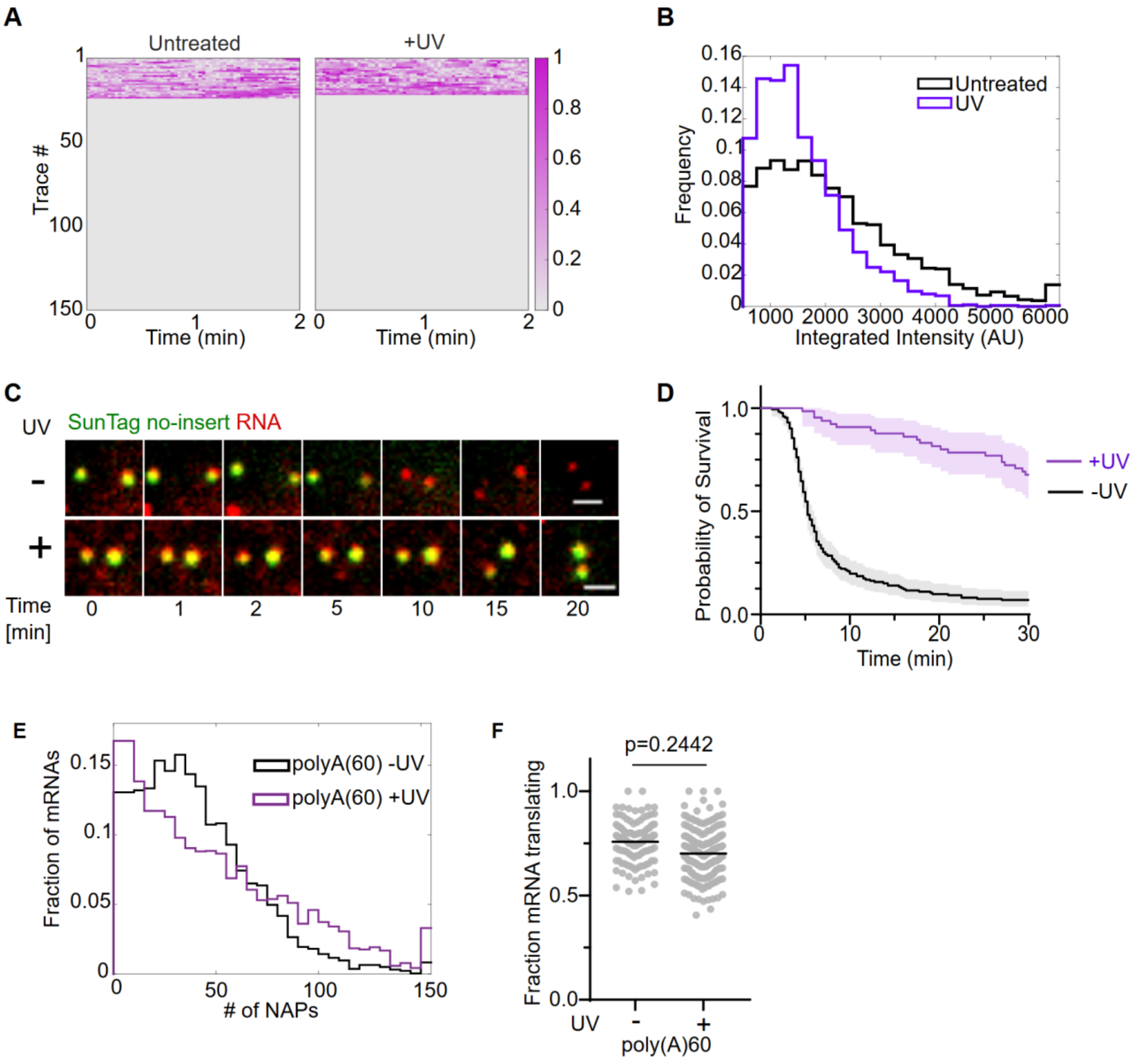

Appendix Figure S6. UV exposure does not increase recruitment of ZNF598 to translating mRNAs, associated with Figure 6.

A. Composite normalized ZNF598-HaloTag intensity at steady state (left) and after UV damage (right) for no-insert mRNAs. (-UV: 13 cells, 150 tracks; +UV: 13 cells, 150 tracks)

B. Integrated intensity of ZNF598-HaloTag spots colocalized to translating mRNA in the no treatment and UV treated conditions for poly(A)60 mRNAs. (-UV: 11, 10 cells, 132, 132 tracks; +UV: 12, 13 cells, 37, 52 tracks)

C. Snapshots from Supplementary Movie 11 of ribosome runoff experiment +/-UV exposure. The time reported was after adding harringtonine. Scale bar: 1μm.

D. The cumulative survival probability of no-insert translation sites on mRNA +/-UV exposure. Cumulative survival probability curve in normal no-insert conditions from Fig. 4D were reproduced here for comparison. Shaded area: 95% confidence bounds computed using Greenwood's formula. 5-9 cells; 65-204 mRNAs were used for each condition.

E. Quantification of the number of Nascent Peptides (NAPs) per mRNA in +/-UV conditions. Mean: -UV= 40.34; +UV= 48.06; Data compiled from two independent experiments. mRNAs calculated to have >150 NAPs are included in the rightmost bin. -UV:117 cells;+UV=184 cells; -UV:5863 mRNAs; +UV:7631 mRNAs per condition; p-value for comparison of +/-UV: 6.4×10^{-12}. P values calculated by two-sample t test after correction of distribution skewness (see methods for details)

F. Fraction of mRNAs actively translating. Each dot represents one cell; black lines indicate mean. p value calculated by two-sample t test.

Appendix Supplementary tables

Appendix Table S1. Sequences of sgRNA DNA template and tracrRNA.

**Name**	**gRNA DNA Sequence (5' to 3')**
ZNF598 gRNA DNA template	CGCTAATACGACTCACTATAgTCACGTAGCTCCCGCCAGCGGTTTAAG AGCTATGCTGGAAACAGCATAGCAA
**Name**	**tracrRNA Sequence (5' to 3')**
tracrRNA	AGCAUAGCAAGUUAAAAUAAGGCUAGUCCGUUAUCAACUUGAAAA AGUGGCACCGAGUCGGUGCUUU

Note: sgRNA DNA template and tracrRNA were ordered and purchased from IDT.

Appendix Table S2. Sequences of DNA primers.

**Name**	**Primer DNA sequence (5' to 3')**
Genotyping FWD Primer ZNF598	AGAAGCCTCTGAGCACCAAG
Genotyping REV Primer ZNF598	AGAAGCCTCTGAGCACCAAG
Sequencing Primer REV Halo	GTGCCATCGCGCGGACCAACA
FWD Amplification primer of sgRNA DNA	CGCTAATACGACTCACTATA
REV Amplification primer of sgRNA DNA	TGCTGGAAACAGCATAGCAAGTTTAAATA AGGCTAGTCCGTTATCAACTTGAAAAAGT GGCACCGAGTCGGTGC
FWD Left Homology Arm-GS Linker-Primer for Donor DNA amplification	TCCCCTCCCTGCAAGCCATCGCCAGGATCA TCACGGGCGGAGGTGGAAGTGGAGGAGGA GGTAGTGCAGAAATCGGTACTGGCTTTC
REV Right Homology Arm Primer for Donor DNA amplification	GAAGGACGCTCACGGTGCGACAGCTCTGG CCACGCaGGtGGtAGtTAACCGGAAATCTCC AGAGTAG

Note: All primers were ordered and purchased from IDT.

Appendix Table S3. SunTag_v4-Cy3 smFISH probe sequences

SunTagV4 smFISH Probe Sequences:	Oligo Sequence (5' to 3')
SunTagV4_probe1	ccacttcgttctcaagatga
SunTagV4_probe2	cccttttcagtctagctac
SunTagV4_probe3	aatttttgctcagcaactcc
SunTagV4_probe4	ttctttagtcgtgctacttc
SunTagV4_probe5	tttcgagagtaactcctcac
SunTagV4_probe6	ccacttcgttttcgagatga
SunTagV4_probe7	acttcccttttttaagcgtg
SunTagV4_probe8	tcttggatagtagctcttca
SunTagV4_probe9	acctcgttctcaagatgata
SunTagV4_probe10	cggaacccttcttcaaacgc
SunTagV4_probe11	agttcttcgagagcagttcc
SunTagV4_probe12	gatcccttttttaatcgagc
SunTagV4_probe13	tgaaagtagttcctcaccac
SunTagV4_probe14	cttcgttttcgaggtggtaa
SunTagV4_probe15	ccctgaacctttctttaatc
SunTagV4_probe16	tactcagtaattcttcaccc
SunTagV4_probe17	tttcgatagcaactcttcgc
SunTagV4_probe18	tttttgagcctagcaacttc
SunTagV4_probe19	ttttcgagagcaactcctcg
SunTagV4_probe20	acctcattttccaagtggta
SunTagV4_probe21	tttgctcaataactcctcgc
SunTagV4_probe22	cgcgacttcgttctctaaat
SunTagV4_probe23	ttcgataagagttcttcgcc
SunTagV4_probe24	ctcattttcgaggtggtagt
SunTagV4_probe25	agtggtagttcttgctcaag
SunTagV4_probe26	ttcaatctcgcgacctcatt
SunTagV4_probe27	attcttgctgagcaattcct
SunTagV4_probe28	cgacttcgttctccaaatga
SunTagV4_probe29	cgacttcattttccaagtgg
SunTagV4_probe30	ttgctcaataactcttcgcc
SunTagV4_probe31	ttcgttctccaagtggtaat
SunTagV4_probe32	agttcttcgataagagctcc
SunTagV4_probe33	gcgacttcattctctaagtg
SunTagV4_probe34	ttcttgctcaagagctcttc

SunTagV4_probe35	cacctcattttccaagtggt
SunTagV4_probe36	ttagatagtaactcttcccc
SunTagV4_probe37	cctcgttctcgagatgataa
SunTagV4_probe38	gatagttcttcgacaggagt
SunTagV4_probe39	cctttttaagtcttgcaacc
SunTagV4_probe40	ttactgagtagttcctcacc
SunTagV4_probe41	ttcgttttccaggtggtaat
SunTagV4_probe42	tcctgatcctttcttcaaac
SunTagV4_probe43	cttttgagagcagttcttcg
SunTagV4_probe44	gcaacctcattttccaaatg
SunTagV4_probe45	tgccacttcccttttttaaa
SunTagV4_probe46	tttcgacagaagttcctcac
SunTagV4_probe47	gctacttcattctcgagatg
SunTagV4_probe48	gagccagaaccctttttaag

Note: All Oligos were ordered and purchased from IDT.
