# Supplementary figures and images for: Single-protein/RNA imaging reveals ZNF598 as a limiting factor in resolving collided ribosomes
Source: EMBO J. 2025 Aug 1;44(18):5215–32. doi: 10.1038/s44318-025-00523-z (PMC12436648; doi:10.1038/s44318-025-00523-z)

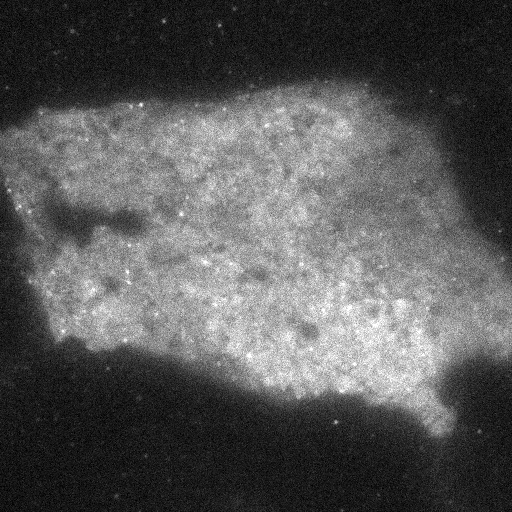

Supplement: Supplementary file 16 — Source data Fig. 1 [file 44318_2025_523_MOESM16_ESM.zip › Figure 1/1C/0xPP7_fullImage.tif]

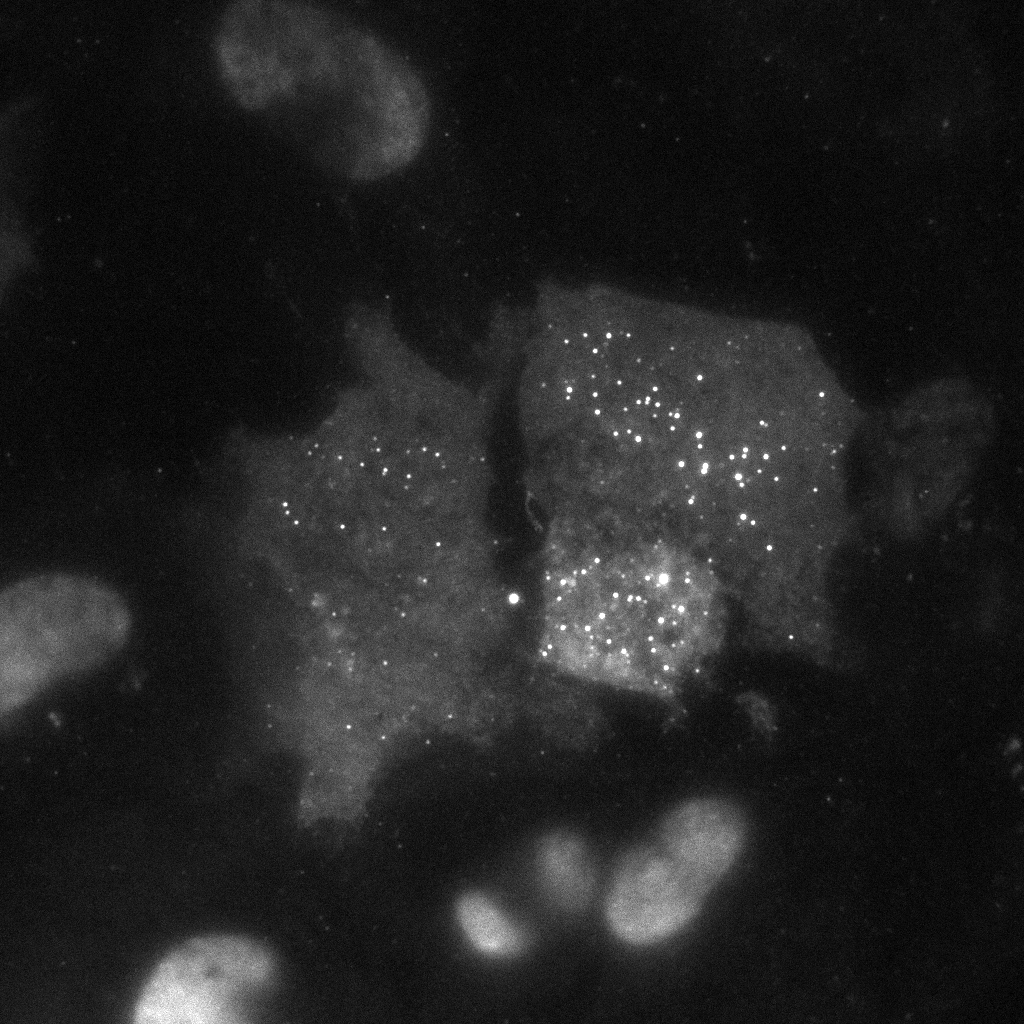

Supplement: Supplementary file 16 — Source data Fig. 1 [file 44318_2025_523_MOESM16_ESM.zip › Figure 1/1C/12xPP7-1_fullimage.tif]

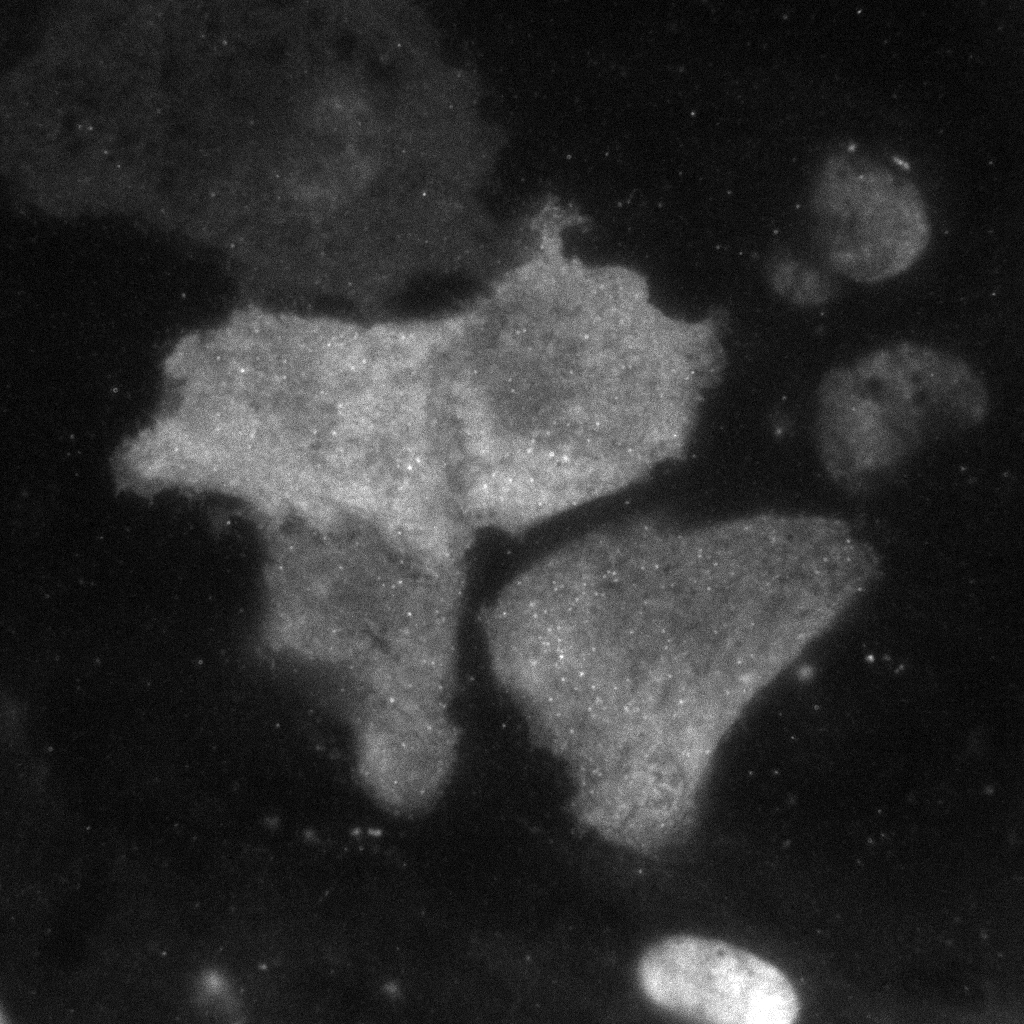

Supplement: Supplementary file 16 — Source data Fig. 1 [file 44318_2025_523_MOESM16_ESM.zip › Figure 1/1C/1xPP7-1_fullimage.tif]

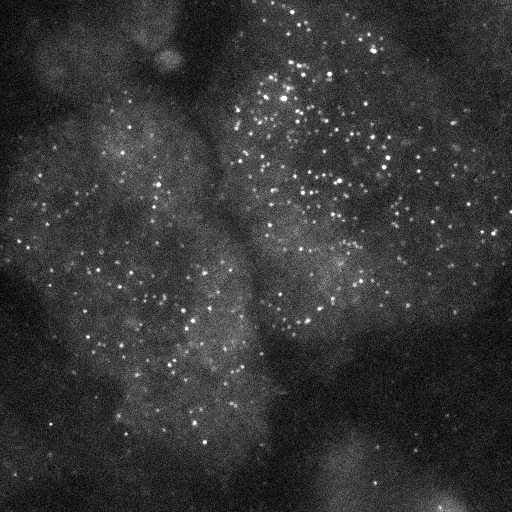

Supplement: Supplementary file 16 — Source data Fig. 1 [file 44318_2025_523_MOESM16_ESM.zip › Figure 1/1C/3xPP7_fullimage.tif]

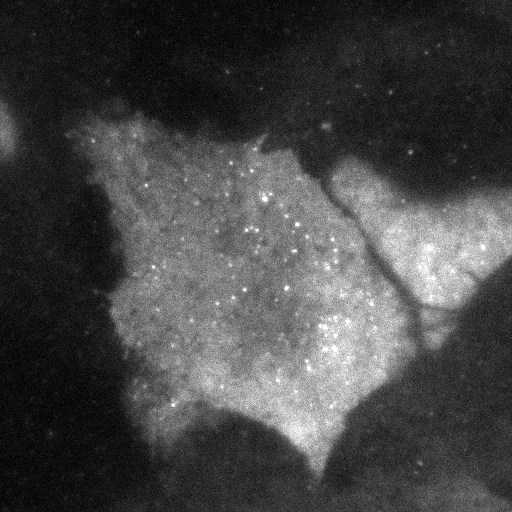

Supplement: Supplementary file 16 — Source data Fig. 1 [file 44318_2025_523_MOESM16_ESM.zip › Figure 1/1C/WholeCellImage.tif]

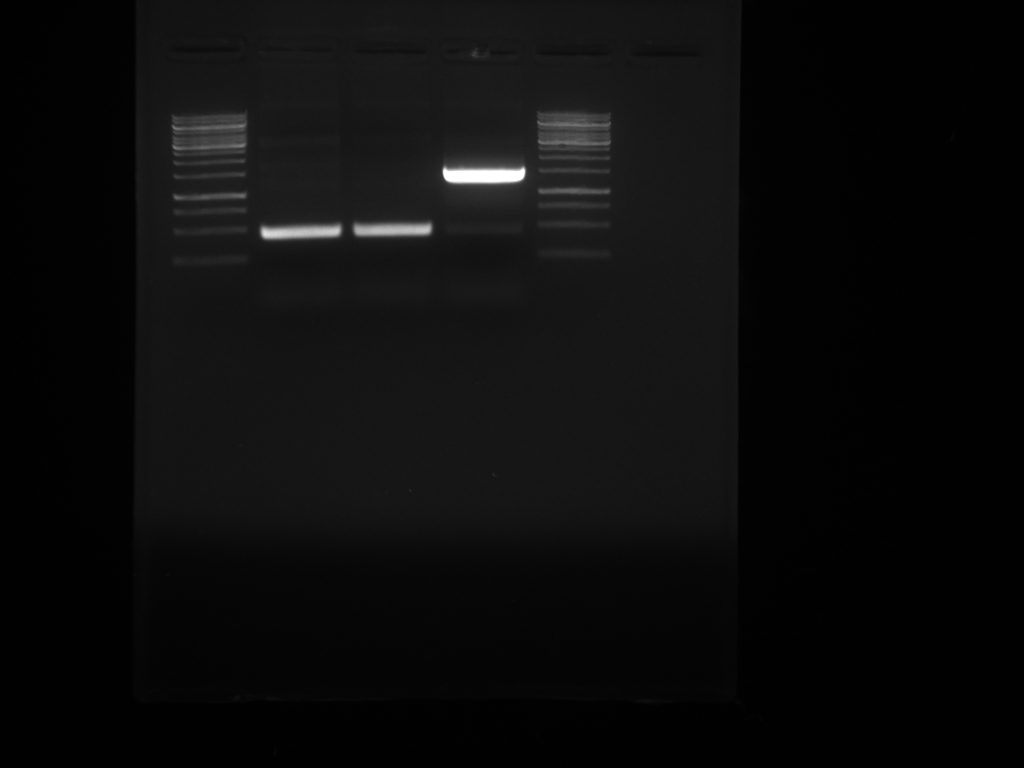

Supplement: Supplementary file 17 — Source data Fig. 2 [file 44318_2025_523_MOESM17_ESM.zip › Figure 2/2B/WT_Knockin_PCR.tif]

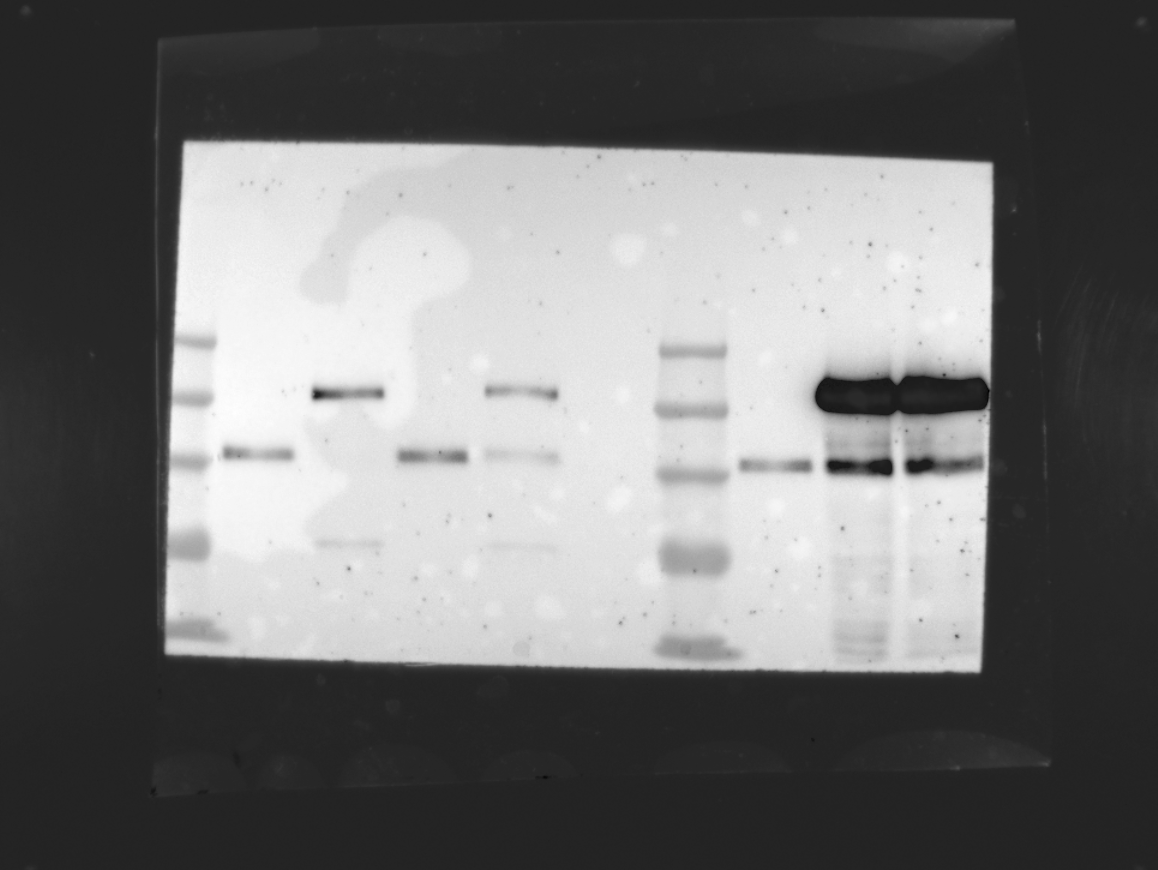

Supplement: Supplementary file 17 — Source data Fig. 2 [file 44318_2025_523_MOESM17_ESM.zip › Figure 2/2C/Knock-In Left _Overexpresion RIGHT.tif]

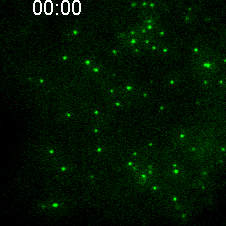

Supplement: Supplementary file 19 — Source data Fig. 4 [file 44318_2025_523_MOESM19_ESM.zip › Figure 4/4C/OE-poly(A)60.tif]

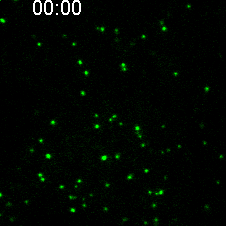

Supplement: Supplementary file 19 — Source data Fig. 4 [file 44318_2025_523_MOESM19_ESM.zip › Figure 4/4C/WT-poly(A)60.tif]

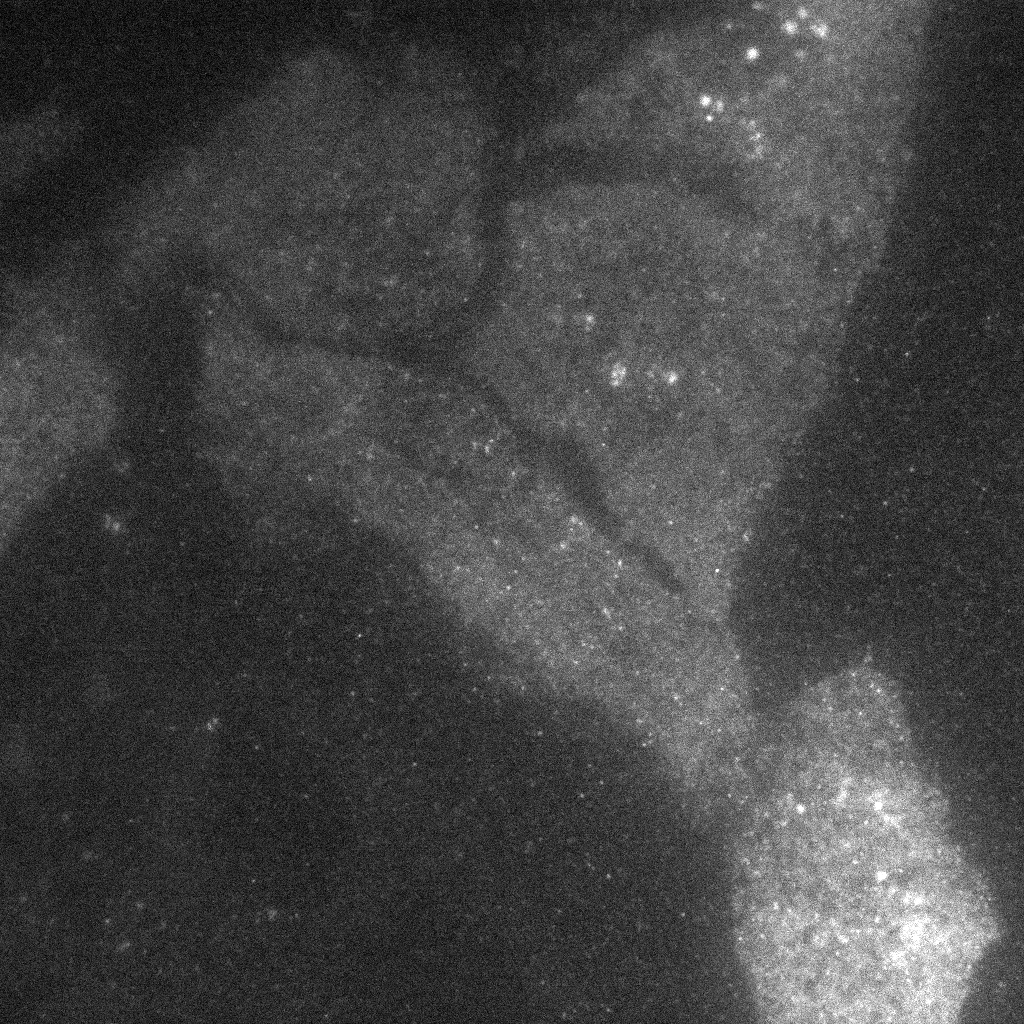

Supplement: Supplementary file 20 — Source data Fig. 5 [file 44318_2025_523_MOESM20_ESM.zip › Figure 5/5B/postHT_Original_Whole_Image.tif]

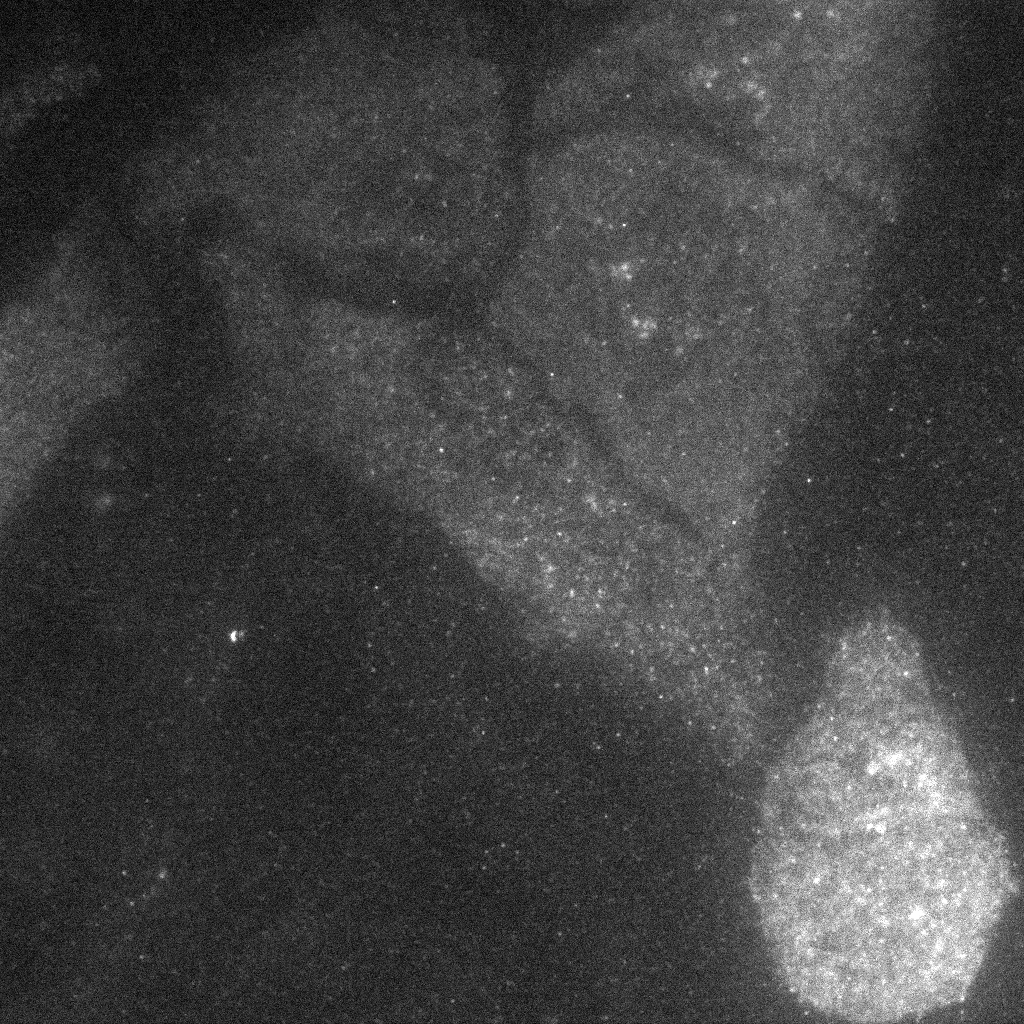

Supplement: Supplementary file 20 — Source data Fig. 5 [file 44318_2025_523_MOESM20_ESM.zip › Figure 5/5B/preHT_Original_Whole_Image.tif]

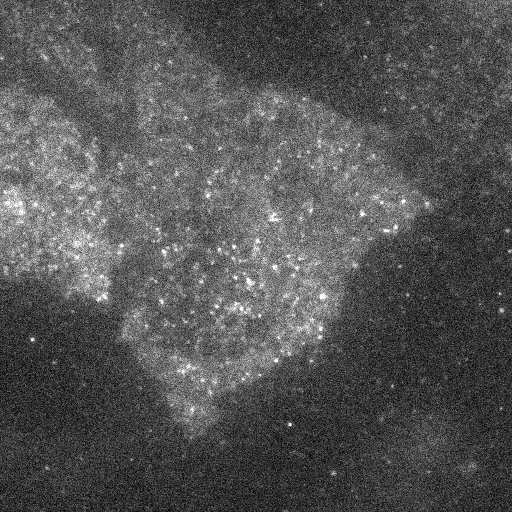

Supplement: Supplementary file 21 — Source data Fig. 6 [file 44318_2025_523_MOESM21_ESM.zip › Figure 6/6B/Original_Image_untreated.tif]
